# Supplementary material for: An Improved Rapid and Sensitive Long Amplicon Method for Nanopore‐Based RSV Whole‐Genome Sequencing
Source: Influenza Other Respir Viruses. 2025 Apr 28;19(5):e70106. doi: 10.1111/irv.70106 (PMC12037990; doi:10.1111/irv.70106)
Supplement: Supplementary file 1 — Table S1. The information for primers used in the modified one‐step multiplex RT‐PCR. Changes highlighted in green have been made to PCR primer concentration and primers of amplifying fragment 3 and 6 compared to our previous publication (1). Table S2. Component for multiplex RT‐PCR. SuperScript IV One‐Step RT‐PCR System with ezDNase (Invitrogen) and primers with concentration at 20 μM are used for the preparation of multiplex PCR mixes below. Table S3. Comparison of sequencing samples with insufficient amplicons by using rapid barcoding (RBK) and rapid PCR barcoding (RPB) kits. NGS results were characterised into four groups including Full‐length (RSV whole genome sequence obtained), G (Full G sequence only), GF (Full F and G sequence obtained) and other (no full G and F sequences obtained). Figure S1. The performance of modified RSV mRT‐PCR for generating RSV genomic amplicons in 135 clinical samples. (A) Histograms of the Ct value distribution for clinical samples with two RSV subgroups, RSV‐A in dark red and RSV‐B in dark green. (B) Tapestation images of RSV amplicons generated from one RSV‐A and one RSV‐B positive clinical samples. DNA marker (M); multiplex RT‐PCR products of pool 1 (lane 1) and pool 2 (lane 2) from an RSV‐A sample; multiplex RT‐PCR products of pool 1 (lane 3) and pool 2 (lane 4) from an RSV‐B sample. Figure S2. RSV NGS achieved by ONT rapid barcoding (RBK) and rapid PCR barcoding (RPB) for samples with insufficient amplicons for NGS library preparation. Coverage depth of sequenced representative RSV‐A (A) and RSV‐B (B) in genomic position with RBK (red) and RPB (blue) for NGS library preparation. [file IRV-19-e70106-s001.docx]

**An improved rapid and sensitive long amplicon method for nanopore-based RSV whole genome sequencing**

Xiaomin Dong^1,2^, Steven Edwards^1^, Yi-Mo Deng^1,2,3^, Clyde Dapat^1^, Arada Hirankitti^1^, Rachel Wordsworth^1^, Paul Whitney^1,2^, Rob Baird^4^, Kevin Freeman^4^, Andrew J Daley^5^, Ian G. Barr ^1,2, #^

**Supplementary Tables**

Supplementary Table 1. The information for primers used in the modified one-step multiplex RT-PCR. Changes highlighted in green have been made to PCR primer concentration and primers of amplifying fragment 3 and 6 compared to our previous publication ([1](#_ENREF_1))

| Multiplex Tube | | Primer Name | Primer Sequence (5’- 3’)^b^ | Position of first 5’ base^a^ | Amplicon length (bp) | Final concentration (μM) |
| --- | --- | --- | --- | --- | --- | --- |
| Tube 1 | 1-1F  1-1R  1-3F3  1-3R6  1-5F  1-5R | | ACGCGAAAAAATGCGTACTACAAAC  CTG**M**ACCATAGGCATTCATAAACA  GCATCACT**W**ACAATATGGGT**K**CC CACTTTTGAT**Y**TTGTTCACTTC**Y**CC TGATGCATCAATATCTCAAGTCA  G**R**CCTAT**D**CCTGCATACTC | 1  1898  3349  6201  7181  11116 | 1898 (fragment 1)  2876 (fragment 3)  3936 (fragment 5) | 0.16  0.16  0.55  0.55  0.6  0.6 |
| Tube 2 | 2-2F  2-2R  2-4F  2-4R1  2-4R2  2-6F  2-6R  2-6R2 | | ATGGGAGA**R**GT**R**GCTCCAGAATA  CGTGTAGCTGT**R**TG**Y**TTCCAA  AGCAAATT**Y**TGGCC**Y**TA**Y**TTTAC  CTCATAGCAACACATGCTGATTG  GAGTTTGCTCATGGCAACACAT  TGGACCAT**W**GAAGC**Y**ATATCA  AGTGTCAAAAACTAAT**R**TCTCGT  ACGAGAAAAAAAGTGTCAAAAACTAAT | 1562  4004  4334  7967  7974  10912  15265  15272 | 2443 (fragment 2)  3634 (fragment 4)  3641 (fragment 4)  4354 (fragment 6)  4365 (fragment 6) | 0.25  0.25  0.6  0.3  0.3  0.44  0.44  0.44 |

^a^Primer nucleotide numbering was based on a human RSV-B strain (GISAID accession numbers 2584506)

^b^Degenerate bases are highlighted in bold.

Supplementary Table 2. Component for multiplex RT-PCR. SuperScript IV One-Step RT-PCR System with ezDNase (Invitrogen) and primers with concentration at 20µM are used for the preparation of multiplex PCR mixes below.

| Component for multiplex PCR mix 1 | Volumes (µL) per reaction |
| --- | --- |
| 1-1F | 0.2 |
| 1-1R | 0.2 |
| 1-3F3 | 0.69 |
| 1-3R6 | 0.69 |
| 1-5F | 0.75 |
| 1-5R | 0.75 |
| 2X Platinum SuperFi  RT-PCR Master Mix | 12.5 |
| SuperScript IV RT Mix | 0.25 |
| template RNA | 8.97 |

| Component for multiplex PCR mix 2 | Volumes (µL) per reaction |
| --- | --- |
| 2-2F | 0.31 |
| 2-2R | 0.31 |
| 2-4F | 0.75 |
| 2-4R1 | 0.38 |
| 2-4R2 | 0.38 |
| 2-6F | 0.55 |
| 2-6R | 0.55 |
| 2-6R2 | 0.55 |
| 2X Platinum SuperFi  RT-PCR Master Mix | 12.5 |
| SuperScript IV RT Mix | 0.25 |
| template RNA | 8.47 |

Supplementary Table 3. Comparison of sequencing samples with insufficient amplicons by using rapid barcoding (RBK) and rapid PCR barcoding (RPB) kits. NGS results were characterised into four groups including Full-length (RSV whole genome sequence obtained), G (Full G sequence only), GF (Full F and G sequence obtained) and other (no full G and F sequences obtained).

| Sample ID | RSV Ct | RSV type | RPB NGS | RBK NGS |
| --- | --- | --- | --- | --- |
| 90007809 | 29 | A | GF | Full-length |
| 90007814 | 30 | B | Other | Full-length |
| 90007817 | 27 | B | G | Full-length |
| 90007819 | 30 | B | G | Other |
| 90008162 | 25 | B | G | Full-length |
| 90008175 | 24 | A | GF | Full-length |
| 90008179 | 26 | A | GF | Full-length |
| 90008178 | 28 | A | G | Full-length |
| 90008183 | 23 | B | GF | Full-length |
| 90008191 | 27 | A | GF | Full-length |
| 90008187 | 27 | A | GF | Full-length |
| 90008196 | 27 | A | GF | Full-length |
| 90008203 | 33 | A | G | Other |
| 90008227 | 28 | B | G | G |
| 90008229 | 32 | A | G | G |
| 90008225 | 27 | A | GF | Full-length |
| 90008228 | 28 | A | G | Full-length |
| 90008224 | 30 | A | GF | G |
| 90008231 | 26 | A | G | Full-length |
| 90008380 | 35 | A | Other | Other |

**Supplementary Figure**

**
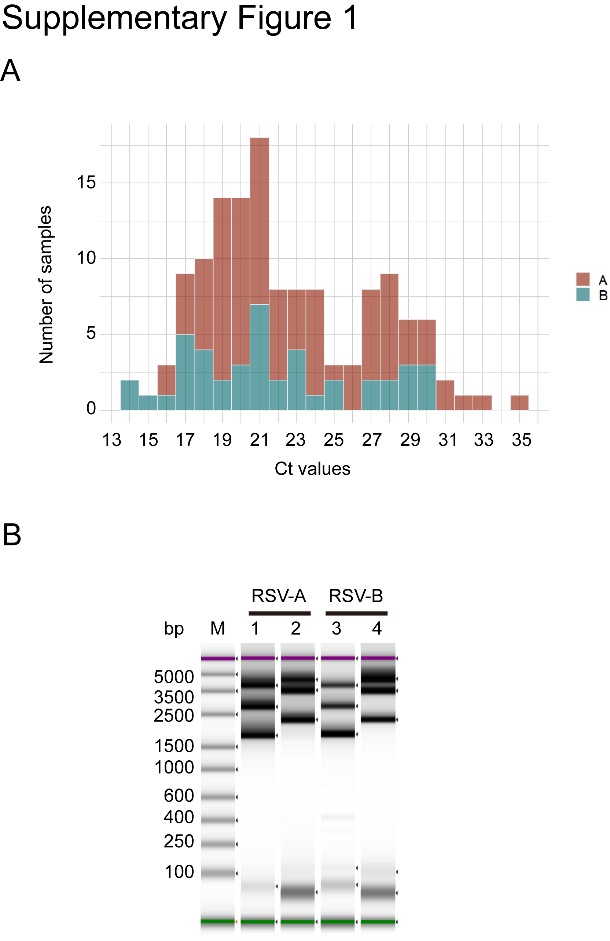
**

Supplementary Figure 1. The performance of modified RSV mRT-PCR for generating RSV genomic amplicons in 135 clinical samples. (A) Histograms of the Ct value distribution for clinical samples with two RSV subgroups, RSV-A in dark red and RSV-B in dark green. (B) Tapestation images of RSV amplicons generated from one RSV-A and one RSV-B positive clinical samples. DNA marker (M); multiplex RT-PCR products of pool 1 (lane 1) and pool 2 (lane 2) from an RSV-A sample; multiplex RT-PCR products of pool 1 (lane 3) and pool 2 (lane 4) from an RSV-B sample.


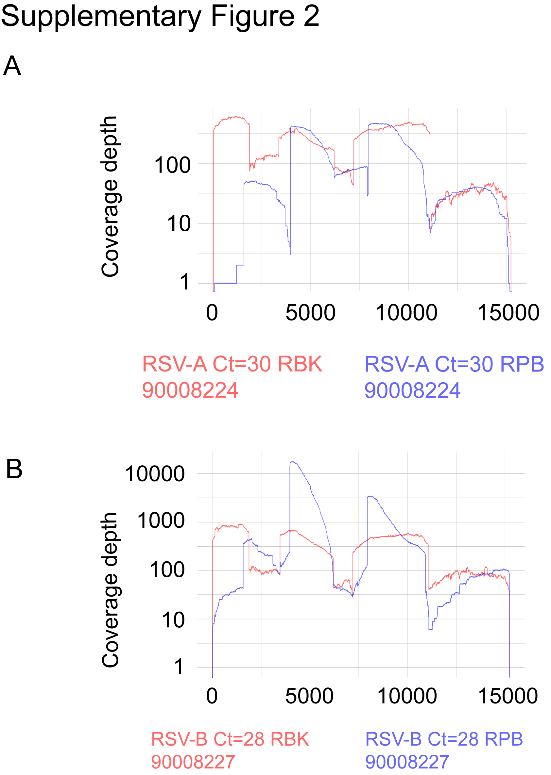


Supplementary Figure 2. RSV NGS achieved by ONT rapid barcoding (RBK) and rapid PCR barcoding (RPB) for samples with insufficient amplicons for NGS library preparation. Coverage depth of sequenced representative RSV-A (A) and RSV-B (B) in genomic position with RBK (red) and RPB (blue) for NGS library preparation

**References**

1. Dong X, Deng YM, Aziz A, Whitney P, Clark J, Harris P, et al. A simplified, amplicon-based method for whole genome sequencing of human respiratory syncytial viruses. J Clin Virol. 2023;161:105423.
